# Combination of Technologies in Body Contouring: Evidence Mapping and a Decision-Making Algorithm

**DOI:** 10.7759/cureus.116270

**Published:** 2026-09-15

**Authors:** Igo Reichmann Losso, Luiz Augusto Sousa Oliveira, Camila Araújo Gomes, Nélio Nunes Cabette Filho, Victor da Costa Sacksida Valladão, Ligia Helena Mendes, Nathalí Eureka Bencosme Veras, Joel Abreu Collado

**Affiliations:** 1 Plastic Surgery, Hospital Niterói D'Or, Serviço de Pós-Graduação em Cirurgia Plástica Prof. Ronaldo Pontes, Niterói, BRA; 2 General Surgery, Fundação Hospitalar Santa Terezinha de Erechim (FHSTE), Erechim, BRA; 3 General Surgery, Hospital Niterói D'Or, Serviço de Pós-Graduação em Cirurgia Plástica Prof. Ronaldo Pontes, Niterói, BRA

**Keywords:** body contouring, cryolipolysis, electric stimulation therapy, high-intensity focused ultrasound ablation, lipectomy, radiofrequency therapy

## Abstract

Body contouring has evolved from a mainly surgical discipline into a multimodal field integrating suction lipectomy, energy-based devices (EBDs), pharmacologic adjuncts, and electromagnetic muscle stimulation. Applying several technologies to the same patient, sequentially or simultaneously, has outpaced the comparative evidence needed to guide their use, leaving surgeons without a structured framework for indication. This narrative review appraises the evidence by tissue target, adipose reduction, cutaneous retraction, and muscle definition, profiles each contemporary technology, and proposes a pragmatic decision-making algorithm anchored in the patient's clinical profile rather than a device hierarchy. Surgical-energy pairings, chiefly radiofrequency-assisted and helium plasma-assisted liposuction, show the most consistent additive skin retraction with acceptable safety. Non-invasive pairings, such as cryolipolysis with high-intensity focused electromagnetic (HIFEM) stimulation and synchronized radiofrequency-HIFEM, produce measurable but modest gains in fat and muscle tone. Pharmacologic adjuncts occupy a narrow niche. Separately, the growing population reshaped by glucagon-like peptide-1 (GLP-1) receptor agonists, presenting skin redundancy with disproportionate lean-mass loss, is emerging as a distinct candidate in whom excision remains primary and muscle-directed therapy gains a physiological rationale. Heterogeneous outcome metrics, a predominance of industry-sponsored series, and the absence of standardized sequencing protocols remain the principal limitations. Technologies are best paired through complementary tissue targets rather than redundant addition; the proposed algorithm stratifies candidates by adipose excess, cutaneous laxity, muscle tone, and scar tolerance to support a rational, individualized choice among monotherapy, sequential non-invasive strategies, and surgical-plus-EBD approaches.

## Introduction and background

What was once an essentially excisional discipline has become a layered specialty in which the surgical bed coexists with a heterogeneous set of devices and pharmacologic adjuncts. Within little more than a decade, modalities as different as controlled adipocyte cooling, focused ultrasound, several iterations of radiofrequency (RF), transcutaneous, microneedling, subdermal, and helium plasma-driven, supramaximal electromagnetic muscle contraction, focused shock waves, selective 1,060 nm diode-laser lipolysis, and injectable bile salts have crossed the threshold of clinical maturity [1-3]. Patients rarely present with an isolated complaint solvable by a single tool; the typical demand mixes localized fat, ptosis of the skin envelope, and a perceived loss of muscular definition. Manufacturers have answered this complexity by promoting device pairings whose biological rationale, different targets and different mechanisms, is plausible, even when comparative trials supporting the specific pairing are scarce or industry-funded [4]. The surgeon is left aligning expectations shaped by marketing with a body of evidence built largely of small prospective series, heterogeneous outcome metrics, and short follow-up.

This narrative review pursues three aims. First, to profile the contemporary technologies relevant to body contouring, indexing them by the tissue compartment they primarily engage rather than by trade name, and summarizing for each its mechanism, indications, expected results, and points of attention. Second, to map what has been published over the past five to seven years on multimodal strategies. Third, to translate that synthesis into a bedside-applicable algorithm that stratifies candidates along four clinical dimensions: magnitude of adipose excess, degree of cutaneous laxity, baseline muscular tone, and tolerance for a surgical scar.

At the outset, the authors wish to state explicitly that they hold no preference for any particular method, device, or manufacturer. The strategies discussed are presented on their merits and their limitations alike; the choice among them, in real practice, rests on the operating surgeon’s training and experience and on an individualized indication for each patient, not on any intrinsic hierarchy among technologies. This review is therefore intended to inform clinical judgment, not to endorse products.

## Review

Methods

We performed a narrative synthesis of the indexed literature. Searches were run on PubMed, Scopus, and Embase, restricted to January 2019 through March 2026 and complemented by manual screening of the reference lists of every full-text article retrieved. The following terms were joined with Boolean operators: “body contouring,” “combined therapy,” “radiofrequency-assisted liposuction,” “helium plasma,” “cryolipolysis,” “high-intensity focused ultrasound,” “microneedling radiofrequency,” “high-intensity focused electromagnetic,” “synchronized radiofrequency,” “1060 nm diode laser,” “laser-assisted liposuction,” “shock-wave therapy,” “collagenase clostridium histolyticum,” “deoxycholic acid,” “GLP-1 receptor agonist,” “semaglutide,” and “tirzepatide.” Eligibility was deliberately broad, including randomized controlled trials, cohorts, systematic reviews, meta-analyses, and pivotal case series in English, Portuguese, or Spanish, but centered on studies applying two or more technologies to the same patient, concurrently or in a defined sequence; single-technology studies were retained only to characterize a device’s mechanism or safety, and isolated case reports without a comparative component were excluded. Each retained study was appraised for funding source, sample size, follow-up, and outcome measurement, including circumferential tape, ultrasonographic adipose thickness, magnetic resonance volumetry, patient-reported instruments, and adverse events; selection ultimately weighed thematic relevance, methodological soundness, and applicability to plastic-surgery practice. A small set of seminal references predating 2019 was retained to anchor pathophysiologic, anatomical, or device-mechanism claims for which no more recent data exist; where such an older study is the principal source for a device’s efficacy, the limitation is noted explicitly.

Tissue targets in body contouring

The premise that justifies combining technologies is anatomical, not commercial. Body-contouring modalities act, in practice, on three biologically distinct targets: the subcutaneous adipocyte and its perilobular fibrous architecture, the dermal-subdermal collagen matrix, and the underlying striated muscle [5]. Each tool engages predominantly one compartment, and the improvement obtainable by acting on a single target tends to be incremental rather than transformative. Adipocytes are addressed by cold-induced apoptosis (cryolipolysis), focused ultrasonic coagulation (high-intensity focused ultrasound (HIFU)), selective photothermal heating (1,060 nm diode laser), mechanical perturbation by focused shock waves (extracorporeal shock-wave therapy (ESWT)), enzymatic membrane disruption (injectable deoxycholic acid), and direct surgical extraction (suction lipectomy and its energy-assisted variants) [6-8]. The dermal-subdermal collagen network is the target of every RF platform, including transcutaneous monopolar and bipolar, fractional microneedling (e.g., Morpheus8), and subdermal probes introduced during liposuction (radiofrequency-assisted liposuction (RFAL), as well as plasma-driven helium devices; in all of them, controlled thermal injury triggers immediate collagen contraction followed by delayed neocollagenesis [9-11]. Striated muscle is recruited by high-intensity focused electromagnetic (HIFEM) platforms and analogous electromagnetic systems that induce supramaximal contractions and progressive hypertrophy [12]. From this reading follows a simple but easily violated principle: useful combinations pair technologies whose primary targets do not overlap. Stacking two adipocyte-directed devices rarely doubles the result, yet it doubles the cost and roughly doubles the adverse-event burden; pairing tools that act on different compartments is where additive and, in selected reports, possibly supra-additive effects may reasonably be expected, provided the word “synergy” is reserved for claims the evidence can actually support.

Technology profiles

The profiles below summarize, for each contemporary modality, its mechanism of action, principal indications, the results that can realistically be expected, and its points of attention or contraindications, grouped by the tissue compartment each modality primarily engages. These profiles are consolidated in Table 1.

**Table 1:** Profiles of contemporary body-contouring technologies For each modality, the table indicates the primary tissue target, the mechanism of action, the principal indications, the results that can realistically be expected, and the main points of attention or contraindications. The evidence column cites a representative source and is not a formal grading. SAL, suction-assisted liposuction; PAL, power-assisted liposuction; UAL, ultrasound-assisted liposuction; LAL, laser-assisted liposuction; RF, radiofrequency; RFAL, radiofrequency-assisted liposuction; HIFU, high-intensity focused ultrasound; HIFEM, high-intensity focused electromagnetic technology; ESWT, extracorporeal shock-wave therapy; CCH, collagenase clostridium histolyticum; HD, high-definition; VASER, vibration amplification of sound energy at resonance.

Technology	Primary tissue target	Mechanism/physiology	Main indications	Expected results	Points of attention/limitations	Evidence (ref.)
SAL/PAL	Adipose (volume)	Mechanical avulsion and aspiration of fat	Localized/regional fat, preserved skin	Predictable volume reduction	No skin tightening; contour irregularity	[5]
UAL/VASER	Adipose	Ultrasonic emulsification, then aspiration	Fibrous zones; high-definition base	Low complication rate; foundation for RF	Thermal injury if misapplied	[20]
LAL	Adipose + collagen	Intralesional laser lipolysis with subdermal heating	Small areas with mild laxity	Measurable skin tightening vs. control	Thermal burn; modest added value	[21]
RFAL (BodyTite)	Collagen + adipose	Internal electrode heats dermal undersurface during aspiration	Localized fat with moderate laxity	Most pronounced non-excisional retraction	Burns; operator experience required	[9]
Helium plasma RF (Renuvion)	Collagen	Ionized-helium RF for rapid subdermal thermal contraction	Moderate laxity; HD contouring	Moderate skin contraction	Thermal risk; adjunct, not standalone	[11]
Transcutaneous RF (monopolar/bipolar)	Collagen	Impedance heating → contraction + neocollagenesis	Mild superficial laxity	Mild tightening	Multiple sessions; modest effect	[10]
Microneedling fractional RF (Morpheus8)	Collagen + adipose	Depth-adjustable (≤8 mm) fractional RF	Laxity ± small subdermal fat	Moderate laxity improvement	Hyperpigmentation risk; operator-dependent	[10]
Cryolipolysis	Adipose	Cold-induced adipocyte apoptosis (panniculitis)	Pinchable localized fat	~1/5 single-cycle fat-layer reduction	Paradoxical adipose hyperplasia (rare); variable response	[3]
HIFU	Adipose	Focused ultrasonic coagulative necrosis at depth	Abdomen/flank localized fat	Modest circumferential reduction	Transient pain and ecchymosis	[7]
1,060 nm diode laser	Adipose	Selective photothermal hyperthermic lipolysis	Non-pinchable abdomen/flank fat	Reproducible thickness reduction	Self-limited inflammatory response	[15]
ESWT	Adipose/cellulite	Acoustic pulses; microcirculatory and septal effect	Cellulite; adjunct	Modest, adjunctive	Not a primary debulking tool	[17]

Adipose-Directed Modalities

Cryolipolysis exploits the differential susceptibility of adipocytes to cold, inducing panniculitis and delayed apoptosis over the weeks following a controlled-cooling cycle; it suits well-defined, pinchable fat in near-normal-weight patients, yields an average single-cycle reduction of roughly 20% of the treated fat-layer thickness, and is limited by variable individual response and the rare but recognized complication of paradoxical adipose hyperplasia, whose incidence and management have been better characterized in recent series [3,6,13]. HIFU delivers focused acoustic energy that produces circumscribed zones of coagulative adipocyte necrosis at a preset depth while sparing the epidermis; it is indicated for localized abdominal and flank fat, produces modest circumferential reduction, and is associated with transient discomfort and ecchymosis, with a recent systematic review supporting its efficacy for both fat reduction and skin tightening [7,8,14]. The 1,060 nm diode laser achieves selective photothermal adipocyte disruption through hyperthermia sustained across the treatment cycle, with contact cooling protecting the dermis; it addresses non-pinchable abdominal and flank fat and has shown reproducible thickness reduction with a predictable, self-limited inflammatory response [15,16]. ESWT applies acoustic pulses used adjunctively for cellulite and to enhance microcirculation rather than as a primary debulking tool, although the principal evidence for its adjunctive use is now dated and awaits contemporary replication [17]. Injectable deoxycholic acid, a bile-salt cytolytic approved only for submental fat, disrupts adipocyte membranes and has been explored off-label in small body depots through dilution and pain-mitigation protocols, where local swelling and the need for repeated sessions temper its role [18,19].

Collagen-Directed (Cutaneous) Modalities

RF platforms convert tissue impedance into heat, and the resulting controlled thermal injury drives immediate collagen contraction and subsequent neocollagenesis. Transcutaneous monopolar and bipolar systems address mild superficial laxity noninvasively; fractional microneedling RF (e.g., Morpheus8) delivers energy at adjustable depths of up to 8 mm, reaching both the subdermal plane and superficial adipose tissue and thereby addressing laxity and small fat excess in a single pass [10]. Subdermal RFAL (BodyTite) introduces an internal electrode during aspiration to heat the undersurface of the dermis directly, producing the most pronounced non-excisional skin contraction among RF options [9]. Helium plasma-driven RF (Renuvion) ionizes helium to deliver rapid, superficial thermal energy for subdermal tissue contraction [11]. Across these platforms, the expected result is measurable but moderate tightening; the principal points of attention are thermal injury, contour irregularity, and, for subdermal devices delivered beneath a flap, the potential to compromise perfusion.

Surgical Extraction and Surgical-Energy Modalities

Suction-assisted and power-assisted lipectomy remain the reference for predictable volume reduction, though on their own they do not tighten skin [5]. Ultrasound-assisted liposuction (UAL; vibration amplification of sound energy at resonance (VASER)) emulsifies fat before aspiration and, in a third-generation series of 261 cases, produced lower complication rates than traditional liposuction with comparable or superior satisfaction, providing a stable foundation onto which subdermal energy may be added [20]. Laser-assisted liposuction (LAL) applies intralesional laser energy to disrupt adipocytes and heat the subdermis; a blinded split-abdomen study documented measurable skin tightening on the laser-treated side relative to a conventional liposuction control [21]. These platforms are best regarded as the surgical substrate to which energy-based skin contraction is coupled, rather than as competitors to it.

Muscle-Directed Electrostimulation

Electromagnetic and electrical muscle stimulation constitute a distinct and rapidly expanding compartment of body contouring; a comprehensive appraisal of this modality falls outside the scope of the present review, and it is therefore summarized here in outline and situated within the multimodal framework. HIFEM technology induces supramaximal, involuntary muscle contractions that, over a course of sessions, are associated with imaging-documented structural change: an abdominal MRI study reported an approximately 15% increase in rectus abdominis thickness and a roughly 19% reduction in overlying subcutaneous fat [12,22]. More recently, platforms delivering HIFEM synchronized with RF in a single applicator (Emsculpt NEO) have been developed to couple muscle stimulation with thermal adipocyte reduction: porcine histologic work has described adipocyte disruption under the dual stimulus [23], magnetic resonance studies have reported concurrent abdominal fat reduction and muscle thickening [24], and comparative work in the gluteal region has suggested greater muscle contouring than HIFEM alone [25]. Absolute effects remain modest, the durability of muscle gains beyond several months is not well characterized, and most data derive from manufacturer-supported studies, caveats that temper the strength of any recommendation and that bear directly on the treatment decisions discussed below.

Cellulite-Directed Injectable Therapy

For the specific indication of cellulite dimpling, injectable collagenase clostridium histolyticum enzymatically cleaves the fibrous septa responsible for skin tethering, and early clinical-practice experience described improvement of buttock and thigh dimpling [26]. The product marketed for this indication was subsequently withdrawn from commercial distribution, for business rather than safety reasons, which limits its present applicability; it is noted here for completeness and because septal cleavage remains a mechanistically distinct target relevant to multimodal planning.

Evidence by technology pairing

Table 2 maps each strategy to its principal tissue target, the type and magnitude of supporting evidence, and the clinical setting in which it is most defensible; the narrative below follows the same order.

**Table 2:** Evidence-mapping matrix of the principal pairings For each strategy, the table indicates the dominant tissue target, the type of available evidence, the approximate magnitude of clinical effect, and the recommended clinical indication. “Type of evidence” reflects the highest design identified for the specific pairing rather than for the individual technologies. The matrix is a pragmatic clinical aid, not a formal grading of evidence; most data derive from small, frequently industry-sponsored series with heterogeneous outcome measures and short follow-up. RF, radiofrequency; RFAL, radiofrequency-assisted liposuction; HD, high-definition; UAL, ultrasound-assisted liposuction; VASER, vibration amplification of sound energy at resonance; HIFEM, high-intensity focused electromagnetic; MRI, magnetic resonance imaging; HIFU, high-intensity focused ultrasound; ESWT, extracorporeal shock-wave therapy; EBD, energy-based device

Strategy	Primary tissue target	Type of evidence	Magnitude of effect	Principal indication	Ref.
Liposuction + subdermal RF (RFAL/BodyTite)	Adipose + collagen	Retrospective cohorts, case series	Moderate additive skin retraction	Localized fat with moderate laxity; HD	[9,27-29]
Liposuction + helium plasma RF (Renuvion)	Adipose + collagen	Retrospective cohorts	Moderate skin contraction	Moderate laxity; HD contouring	[30-32]
Third-generation UAL (VASER) ± subdermal RF/plasma	Adipose + collagen	Case series	Low-complication base for added retraction	Definition with concurrent laxity	[20]
Cryolipolysis + HIFEM	Adipose + muscle	Open-label studies; systematic review	Small-moderate additive (fat + tone)	Non-invasive fat reduction with toning	[22,35]
Synchronized RF + HIFEM (Emsculpt NEO)	Adipose + muscle	Porcine histology; MRI cohorts	Concurrent fat reduction + muscle gain	Non-invasive fat + tone, single device	[23-25]
Cryolipolysis + HIFU	Adipose	Pilot trial; case reports	Modest; pain attenuation when sequenced	Non-invasive flank/abdominal fat; pain	[36,37]
Microneedling RF (Morpheus8)	Collagen + adipose	Case series; open study	Moderate laxity improvement	Skin laxity ± subdermal fat	[38,39]
Cryolipolysis + focused shock waves (ESWT)	Adipose; cellulite	Small comparative series	Modest additive effect	Cellulite with localized fat	[17]
Subdermal RF + electrical muscle stimulation	Collagen + muscle	Early clinical reports	Additive circumferential reduction + tone	Additive fat, laxity, and tone	[41]
Radiofrequency + aerobic exercise	Adipose	Randomized controlled trial	Additive lipolysis	Adjunct to lifestyle intervention	[40]
Deoxycholic acid (off-label) + EBD	Adipose	Off-label use; expert opinion	Variable	Small residual fat deposits as adjunct	[18]
Excisional surgery + EBD ± HIFEM (post-bariatric/post-GLP-1)	Skin redundancy + lean-mass loss (± residual fat)	Narrative reviews; systematic review/meta-analysis	Surgery dominant; EBD and HIFEM adjunctive	Massive weight loss (bariatric or GLP-1) with severe redundancy	[42,45,46]

Surgical-Energy-Based Pairings

The pairing of liposuction with subdermal RF (RFAL, BodyTite) or with helium plasma-driven RF (Renuvion) is among the most documented surgical-energy strategies. In gynecomastia correction, the addition of RF to power-assisted liposuction was reported to facilitate a more effective chest lift than liposuction alone [27]. In upper-arm RFAL, a series reported durable circumferential reduction with high satisfaction scores [28]. A two-stage RFAL lipoabdominoplasty protocol has been described as safe in selected candidates with moderate cutaneous laxity [29]. Helium plasma-driven RF co-applied with UAL has been examined in retrospective cohorts: in the largest series to date (639 patients), the pairing showed an acceptable safety profile without a significant excess of major adverse events relative to UAL alone [30], and a separate retrospective evaluation of helium plasma-assisted lipoabdominoplasty corroborated this safety signal [31]. In high-definition contouring, helium plasma RF has been integrated for selective subdermal coagulation [32]. Third-generation UAL (VASER) provides the low-complication foundation onto which these energy modalities are layered [20]. A related variant couples liposuction with argon-generated plasma. A single-arm arm-contouring series reported reduced ultrasonographic tissue thickness and improved skin elasticity at six months with only minor seromas [33], and ex vivo work has begun to contrast argon with helium plasma on human skin [34], an emerging option pending controlled comparison. Read across these independent series, different regions, devices, and investigators, the surgical-energy data are notably concordant: added skin retraction and the absence of excess major morbidity recur consistently, making this the most reproducible signal in the multimodal literature, even though effect magnitudes are reported on non-comparable scales.

Non-invasive Pairings

Cryolipolysis co-applied with HIFEM has been examined in open-label studies reporting reduction of subcutaneous fat together with measurable muscle hypertrophy, with effects that appeared additive relative to either modality alone, though comparative confirmation is limited [22]. A critical systematic review of electromagnetic treatments acknowledged a favorable safety profile for HIFEM with or without concurrent RF, but cautioned pointedly that the reported benefits rest largely on industry-funded studies using heterogeneous, frequently surrogate endpoints, and that mean treatment effects are small, typically below 5 mm of circumferential change, a sobering counterpoint to manufacturer claims that underscores the need for realistic expectation-setting [35]. The synchronized delivery of RF and HIFEM in a single applicator has been reported to reduce abdominal fat while increasing muscle thickness on MRI [24], with supporting histologic [23] and regional comparative [25] data. Cryolipolysis followed by HIFU has been described both to enhance adipocyte reduction and to attenuate the discomfort of HIFU delivered to previously cooled tissue [36]; a pilot comparative trial favored HIFU over cryolipolysis as monotherapy for flank fat, although the additive value of the sequence has not been confirmed in randomized designs [37]. Microneedling RF has emerged as a versatile platform addressing subdermal fat and cutaneous laxity through adjustable penetration depth: single-treatment protocols improved arm, submammary, and suprapatellar laxity [38], and an open study addressed abdominal laxity in postpartum women [39]. In contrast to the surgical-energy evidence, the non-invasive literature is internally discordant: independent and industry-supported reports disagree less about the direction of effect than about whether its magnitude, often below 5 mm, is clinically meaningful.

Adjunctive and Pharmacologic Pairings

ESWT has been investigated for cellulite as monotherapy and paired with cryolipolysis through dual probes, with a modest additive effect [17]. RF plus aerobic exercise produced additive lipolytic effects in a randomized design [40]. Subdermal RF co-applied with electrical muscle stimulation was reported to yield additive circumferential reduction with improved muscle tone in early clinical reports [41]. Injectable deoxycholic acid, approved only for submental fat, has been explored off-label in body regions and proposed as a complement to energy-based devices (EBDs) that primarily address skin laxity [18].

Post-bariatric Scenario

In patients after massive weight loss, the dominant tissue defect is cutaneous redundancy disproportionate to residual adiposity. Surgical excision, abdominoplasty, brachioplasty, thigh and lower-body lifts, remains the cornerstone, and EBDs play an adjunctive role for areas not amenable to excision and for refining moderate residual laxity [42]. Substituting excisional procedures with EBDs alone is not supported by current evidence in this subgroup.

The Post-GLP-1 Body-Contouring Patient

A development that postdates much of the multimodal literature has begun to reshape the body-contouring population itself: the widespread use of glucagon-like peptide-1 (GLP-1) receptor agonists, semaglutide and the dual GIP/GLP-1 agonist tirzepatide, for weight reduction. This largely non-surgical weight loss yields a phenotype that overlaps with, but is not identical to, the post-bariatric patient: substantial cutaneous redundancy alongside a disproportionate loss of lean body mass, since a meaningful fraction of the weight lost is skeletal muscle rather than fat [43,44]. The result is a candidate presenting all three tissue targets at once: residual adiposity, a lax skin envelope, and diminished muscle volume, but with a more prominent muscle deficit, prompting explicit calls to adapt contouring strategies to this sarcopenic morphology [45].

Two implications follow. First, excision remains the definitive treatment for the redundant skin envelope, as in the post-bariatric setting; EBDs do not substitute for it when redundancy is severe [45]. Second, the characteristic lean-mass depletion supplies a physiological rationale for muscle-directed therapy: HIFEM and synchronized RF-HIFEM, modest in the general population, become more defensible here as an adjunct aimed at restoring muscle volume rather than reducing fat [44]. Perioperative data are emerging; a meta-analysis found no significant excess of major complications from GLP-1 agonists in body-contouring surgery, though the recognized delay in gastric emptying bears on anesthetic planning [46]; protein-sufficient nutritional optimization to protect lean mass is increasingly advised [47]. The evidence remains young and descriptive, and this review treats the post-GLP-1 patient as an emerging clinical context rather than a validated protocol.

Decision-making algorithm

Translating this evidence into decisions requires a framework built on the four clinical dimensions most consistently tied to technology selection: (a) magnitude of adipose excess (localized, regional, or diffuse); (b) degree of cutaneous laxity (preserved, moderate, or severe); (c) baseline muscular tone (preserved or deficient, including rectus diastasis); and (d) tolerance for a surgical scar (excisional acceptable, minimally invasive only, or strictly non-invasive). Table 3 sets out the resulting strategy for each dominant clinical pattern, and Figure 1 renders the full pathway.

**Table 3:** Decision framework by dominant clinical profile Quick-reference companion to the algorithm in Figure 1. The table pairs each dominant clinical profile with a preferred primary strategy and rational add-ons for concurrent findings; it is intended to structure clinical judgment, not to replace individualized indication. RF, radiofrequency; RFAL, radiofrequency-assisted liposuction; HIFU, high-intensity focused ultrasound; HIFEM, high-intensity focused electromagnetic technology; ESWT, extracorporeal shock-wave therapy; EBD, energy-based device

Dominant clinical profile	Preferred primary strategy	Rational add-on(s)
Localized fat, skin preserved, scar acceptable	Traditional or ultrasound-assisted liposuction	Subdermal RF/helium plasma if moderate laxity or high-definition is sought
Localized fat with moderate laxity, scar acceptable	Liposuction + subdermal RF (RFAL) or helium plasma	Microneedling RF for residual superficial laxity
Severe cutaneous laxity (including post-bariatric)	Excisional surgery (abdominoplasty/brachioplasty/body lift)	EBD to refine non-excised areas; avoid EBD substitution
Localized fat, minimally invasive preference	Cryolipolysis, HIFU, or 1,060 nm diode laser	Sequential HIFEM (or synchronized RF-HIFEM) for tone; microneedling RF for laxity
Muscular hypotonia with limited adiposity	HIFEM-based therapy (± synchronized RF)	Single non-invasive modality for minor residual fat
Cellulite with localized fat	ESWT ± cryolipolysis	Targeted septal therapy where appropriate

**Figure 1:**
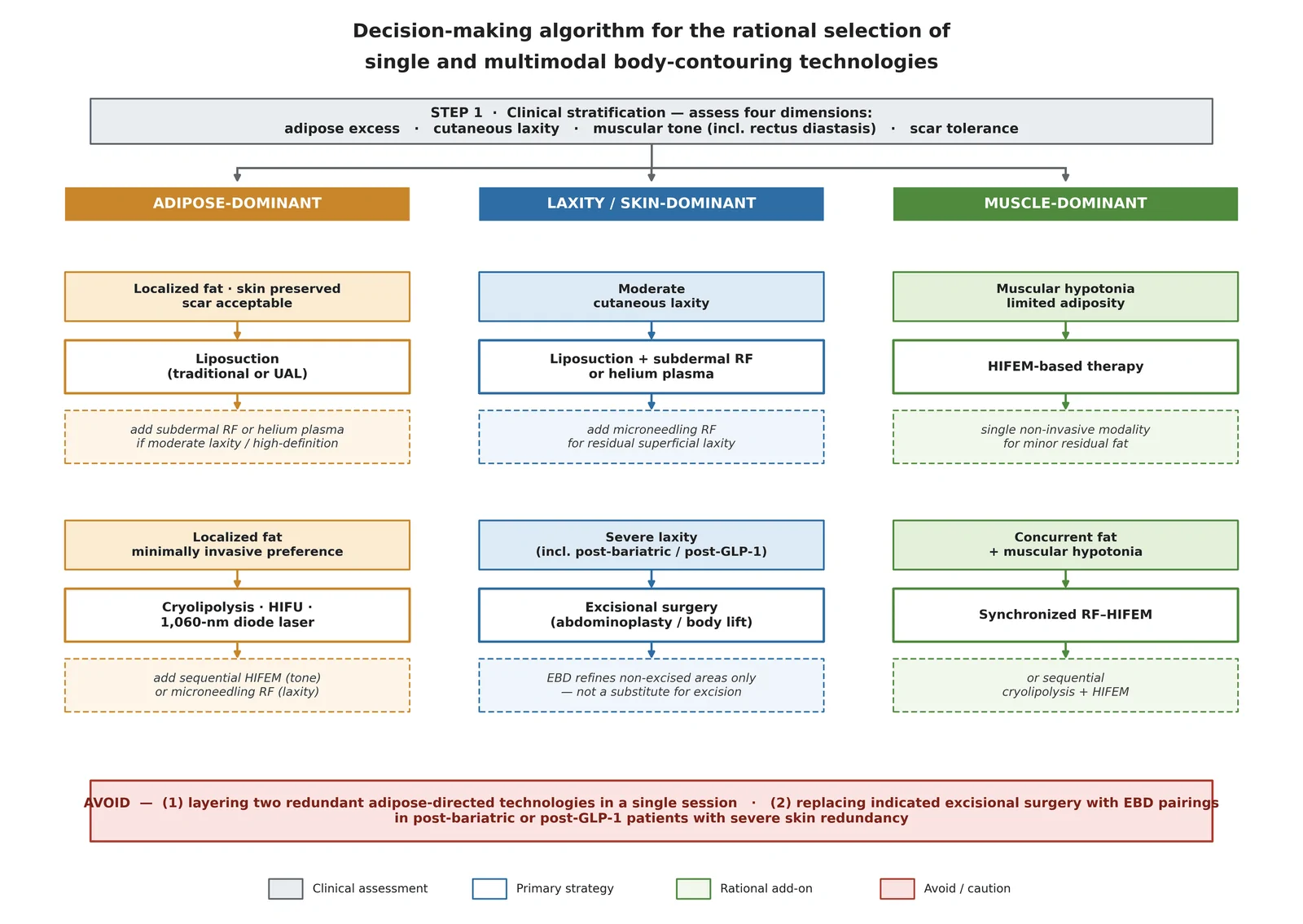
Decision-making algorithm for the rational selection of single and multimodal body-contouring technologies The algorithm stratifies candidates by four clinical dimensions: magnitude of adipose excess, degree of cutaneous laxity, baseline muscular tone, and tolerance for a surgical scar, and guides selection among monotherapy, sequential non-invasive strategies, and surgical-plus-EBD approaches. It also flags the post-bariatric and post-GLP-1 patient as a special scenario in which excision cannot be replaced by EBDs. RF, radiofrequency; UAL, ultrasound-assisted liposuction; HIFU, high-intensity focused ultrasound; HIFEM, high-intensity focused electromagnetic technology; GLP-1, glucagon-like peptide-1; EBD, energy-based device Image credits: Original figure created by the authors with Matplotlib (Python), based on the evidence synthesized in this review.

In localized adipose excess with preserved skin and acceptable scar tolerance, traditional or UAL remains the most predictable single intervention; subdermal RF or helium plasma is added when moderate laxity is present or a high-definition result is sought. With severe laxity, excisional procedures take precedence and EBDs serve to refine non-excised areas. In localized adiposity with a minimally invasive preference, cryolipolysis, HIFU, or the 1,060 nm diode laser constitute the primary strategy; sequential HIFEM or synchronized RF-HIFEM addresses concurrent muscular hypotonia, and sequential microneedling RF addresses concurrent moderate laxity. When the dominant complaint is muscular hypotonia with limited adiposity, HIFEM-based therapy is the primary indication. The framework explicitly discourages layering two redundant adipocyte-directed technologies in one session and substituting clearly indicated excisional surgery with EBD pairings in post-bariatric patients with severe redundancy.

Discussion

Quality of the Evidence Base

Although publications on multimodal body-contouring strategies have grown considerably since 2019, the internal quality of this literature remains uneven, and several recurring weaknesses deserve to be made explicit. The first is structural: industry-sponsored prospective series dominate the available evidence, while randomized comparisons against an active comparator remain rare, which limits any attempt at head-to-head ranking of these strategies. The second is methodological: outcome metrics vary widely across studies, including tape circumference, ultrasonographic fat thickness, magnetic resonance volumetry, and an assortment of validated and ad hoc satisfaction scales, which precludes meaningful pooled analysis. The third concerns protocol design: sequencing rules, including which modality is used first, what interval is used between sessions, and which criteria trigger retreatment, are rarely standardized and often go unreported. The fourth is duration: most series stop at six months, a window too narrow to capture delayed neocollagenesis, late paradoxical adipose hyperplasia, or contour evolution after paired surgical-energy procedures. The sharpest expression of these concerns comes from a critical systematic review of electromagnetic body contouring, which questioned whether the small, largely industry-derived effect sizes reported for these devices are clinically meaningful at all, a caution that should be extended to multimodal claims generally [35].

Consistency and Divergence Across Studies

Set against these limitations, the evidence is not uniformly uncertain, and separating what is reproducible from what is contested is itself a practical output of this review. The most consistent signal is the additive cutaneous retraction of surgical-energy pairings: independent series using RF-assisted or helium plasma-assisted liposuction, across different regions and the largest safety cohort to date, agree on both the direction of benefit and the absence of excess major morbidity [28,30,31]. The sharpest disagreement surrounds non-invasive electromagnetic devices, where manufacturer-supported trials and an independent critical appraisal concur that fat and muscle change in the expected direction yet diverge on whether the sub-5 mm magnitude is clinically meaningful [24,35]. An intermediate, unresolved question is the relative value of cryolipolysis and HIFU for the same adipose depot, where a head-to-head pilot favored HIFU as monotherapy while the rationale for combining them rests on uncontrolled sequential data [36,37].

Safety of Stacked Energy Delivery

The thermal load delivered by two energy devices in close temporal sequence does not behave linearly; panniculitis, dermal burns, and contour irregularities may emerge from pairings that, taken individually, would be unremarkable. Paradoxical adipose hyperplasia after cryolipolysis remains uncommon but well documented, and recent series have refined its expected incidence and surgical management [13]. Adding subdermal RF or helium plasma to an immediate excisional procedure may, depending on the anatomical territory, compromise flap perfusion. Cost-effectiveness, central to real-world decision-making, has been almost entirely absent from the published reports.

Limitations and Future Directions

Despite these caveats, the framework of complementary tissue targets remains a coherent way to separate pairings that follow a biological logic from those that follow a commercial one. The algorithm we propose is deliberately minimal, with four clinical axes and a small number of branches, so that it can be used at the bedside without hardening into a rigid checklist; it is meant to structure clinical judgment, not to replace it, and, consistent with the position stated at the outset, it privileges no device or manufacturer. This review has limitations of its own: it is narrative rather than systematic, no formal risk-of-bias appraisal or quantitative synthesis was performed, and the proposed algorithm reflects the current evidence base and expert reasoning rather than prospective validation. Future work should converge on randomized comparative trials with standardized imaging endpoints, transparent sequencing protocols, follow-up extending well beyond six months, and explicit reporting of cost.

## Conclusions

In body contouring, technologies are best paired through complementary tissue targets, adipocyte, dermal-subdermal collagen, and striated musculature, rather than through redundant aggregation of devices acting on the same compartment. Surgical-energy pairings (RF-assisted and helium plasma-assisted liposuction) currently offer the most consistently reported evidence of additive cutaneous retraction with acceptable safety. Non-invasive pairings, including cryolipolysis with HIFEM and the synchronized delivery of RF with HIFEM, produce smaller but measurable additive effects on fat and muscle tone. Excisional procedures remain irreplaceable in patients with severe cutaneous redundancy, particularly after massive weight loss and in the growing population reshaped by GLP-1 receptor agonists, and should not be substituted by stacked EBDs. The proposed decision-making algorithm stratifies candidates according to four clinical dimensions and provides a practical framework for rational, individualized indication. Methodologically robust comparative trials remain necessary to refine sequencing and consolidate the evidence base.
